# DOPAMAP, high-resolution images of dopamine 1 and 2 receptor expression in developing and adult mouse brains

**DOI:** 10.1038/s41597-022-01268-8

**Published:** 2022-04-19

**Authors:** I. E. Bjerke, E. R. Cullity, K. Kjelsberg, K. M. Charan, T. B. Leergaard, J. H. Kim

**Affiliations:** 1 Neural Systems Laboratory, Institute of Basic Medical Sciences, University of Oslo, Oslo, Norway; 2 Mental Health Theme, Florey Institute of Neuroscience and Mental Health, Melbourne, Australia; 3 ISN Psychology, Institute for Social Neuroscience, Ivanhoe, Australia; 4 IMPACT – the Institute for Mental and Physical Health and Clinical Translation, School of Medicine, Deakin University, Geelong, VIC Australia

**Keywords:** Molecular neuroscience, Neuronal development

## Abstract

The dopaminergic system undergoes major reorganization during development, a period especially vulnerable to mental disorders. Forebrain neurons expressing dopamine 1 and 2 receptors (D1R and D2R, respectively) play a key role in this system. However, neuroanatomical information about the typical development of these neurons is sparse and scattered across publications investigating one or a few brain regions. We here present a public online collection of microscopic images of immunohistochemically stained serial sections from male and female mice at five stages of development (postnatal day 17 (P17), P25, P35, P49, and adult), showing the distribution of D1R and D2R expressing neurons across the forebrain. All images from adult brains are registered to the Allen Mouse brain Common Coordinate Framework, while images from P17-P35 age groups are registered to spatially modified atlas versions matching the morphology of young brains. This online resource provides microscopic visualization of the developing dopaminergic system in mice, which is suitable as a benchmark reference for performing new experiments and building computational models of the brain.

## Abstract (summary)

**Table:** 

Measurement(s)	mRNA expression
Technology Type(s)	transgenic Mouse • immunohistochemistry staining method
Sample Characteristic - Organism	Mus musculus

## Background & Summary

From infancy through adolescence and into adulthood, the brain undergoes major development and reorganization. Such changes are also associated with the onset of mental disorders such as anxiety, depression, bipolar disorder, and schizophrenia, which rank among the leading causes of disease burden worldwide^1,2^. Changes in the dopaminergic system are particularly evident during adolescence, and have been proposed to contribute to the increased vulnerability to mental disorders seen during this period^3^.

Dopaminergic cells located in the substantia nigra and ventral tegmental area project to several forebrain regions^4^. These dopaminergic inputs reach neurons expressing specific dopamine receptors, with the most common types being the dopamine 1 and 2 receptors (D1R and D2R)^5^. These receptors distinctively contribute to several higher cognitive functions such as attention, goal-directed behavior, reward processing, and memory^6–9^. To discern the putative role of dopamine in the ontogeny of different mental disorders, we need a detailed understanding of the typical development of the dopaminergic system.

Neuroanatomical information describing the dopamine receptor positive neurons have so far been sparse, with studies impeded by the lack of sensitive and specific antibodies for these receptors^10^. More recently, transgenic animals have proved useful for quantification of D1R and D2R cells^11,12^. In particular, Cullity and colleagues performed a stereological study to quantify the cells expressing these two receptors in five regions of the rostral forebrain^10^. This investigation has generated a substantial body of histological data that is useful for addressing hypotheses beyond those addressed in the original publication.

**Fig. 1:**
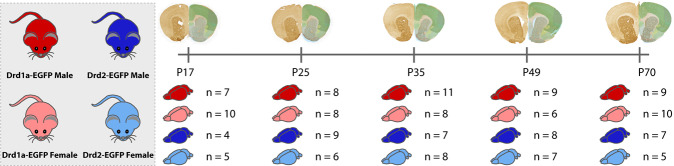
Overview of the DOPAMAP data. The DOPAMAP collection contains high-resolution microscopic images of immunohistochemically stained sections. The datasets span two genotypes (Drd1a- and Drd2-EGFP, shown in red and blue, respectively), both sexes and five age groups (from P17 to P70), combined in a total of 20 datasets, illustrated with genotypes and number of subjects indicated for each age group. All the data have been spatially registered to the Allen Mouse brain Common Coordinate Framework. The top right panel shows example microscopic images from the different age groups with atlas overlay shown on the right hemisphere.

We here present the comprehensive collection of immunohistochemically-stained material used in the study by Cullity and colleagues^10^. While the previous study used the collection to quantify cells in five brain regions and showed example images of the sections, the current study digitizes and presents the entire collection as an online resource of high-resolution images. Thus, we here make this valuable collection available to all researchers, preparing and preserving the material for studies beyond those presented by Cullity and colleagues^10^. The shared data include serial sections from 79 male and 74 female mice at five stages of postnatal development (P17, P25, P35, P49, and adult), showing the distribution of D1R and D2R expressing neurons across the forebrain of the developing mouse. An overview of the transgenic animals and age groups included in the different datasets is given in Fig. 1. All images are spatially registered to the Allen Mouse brain Common Coordinate Framework^13^. For the P17-P35 age groups, versions of the atlases that have been modified to match the morphology of young brains^14^ were used. The high-resolution images, shared via the EBRAINS Knowledge Graph (https://search.kg.ebrains.eu/), can be downloaded or interactively inspected with atlas overlays using an online microscopic viewer. This public data collection of microscopic images showing the developing dopaminergic system in mice is highly suitable as a benchmark reference for experiments using mouse models of dopamine-related disorders.

## Methods

The datasets presented here include microscopic images of coronal sections from Drd1a-EGFP and Drd2-EGFP mice, immunohistochemically stained to reveal EGFP expression in the cells expressing dopamine 1 receptors (D1R) and dopamine 2 receptors (D2R), respectively. The data were generated and partially analyzed by Cullity and colleagues^10^. Male and female mice at five ages corresponding to key cognitive-affective development milestones are included: juvenile (postnatal day (P) 17); preadolescent (P25), adolescent (P35), late adolescent (P49), and adult (P70)^15^. The data are sorted into 20 datasets, each representing one combination of genotype, sex and age. The number of animals within each dataset is listed in the Data Records section. Below, we first describe the animals used and the immunohistochemistry performed, before detailing the methods for digitizing sections, pre-processing images, and spatially registering images to age specific reference atlases.

### Animals and immunohistochemistry

Experimental procedures involving animals were approved by the Florey Institute of Neuroscience and Mental Health Animal Ethics Committee and performed in accordance with the Australian code for the care and use of animals for scientific purposes (Eight Edition^16^). Drd1a-EGFP (RRID:MMRRC_000297-MU) and Drd2-EGFP (RRID:MMRRC_000230-UNC) mice were used, originally generated through the Gene Expression Nervous System Atlas (GENSAT) program^17^ (Rockefeller University, New York, USA) and bred on an Arc:arc Swiss background at the Florey Institute of Neuroscience and Mental Health, Melbourne, Australia. Animals were weaned at P18–21, except P17 groups that were weaned 30 minutes prior to perfusion. At weaning, mice were housed in groups of 3–5 animals of the same sex in open-top cages (34 cm × 16 cm × 16 cm). Animals were maintained in a temperature-controlled environment on a 12 hour light/dark cycle (lights on from 07:00), with food and water available ad libitum.

On postnatal day (P) 17, 25, 35, 49 or 70, animals were transcardially perfused while under sodium pentobarbitone anesthesia (100 mg/kg given intraperitoneally). Mice were first perfused with 50 mL 0.1 M phosphate buffered saline (PBS), followed by 50 mL 4% paraformaldehyde (PFA) in phosphate buffer (PB). Brains were extracted, post-fixed for 1 hour in 4% PFA, and stored overnight at 4 °C in a solution of 20% w/v sucrose in PB. Brains were then snap frozen using liquid nitrogen and stored at −20 °C until sectioning. To allow identification of left and right hemispheres, a needle was inserted through the left hemisphere, after which the brains were cut coronally into 40 µm thick sections on a cryostat (Leica, Germany). Sections were stored in a cryoprotective solution until they were processed for immunohistochemistry.

Every fourth section was used for free-floating immunohistochemistry according to the avidin-biotin method. Unless stated otherwise, all steps were performed on an orbital shaker at room temperature. Sections were washed with PBS for 3 × 10 minutes, before 15 minutes of incubation in 10% H_2_O_2_ and 10% methanol in PBS to quench endogenous peroxidase activity. After washing in PBS for 3 × 5 minutes, sections were incubated overnight with the primary antibody, chicken anti-GFP (RRID:AB_300798), diluted 1:1000 in a blocking solution of 0.5% normal goat serum and 0.5% triton X-100 (TX-100) in PBS. The next day, sections were first washed 3 × 5 minutes in PBS before incubation for 30 minutes with the blocking solution (10% normal goat serum and 0.3% Triton X-100 in PBS). Sections were then incubated for 1 hour with the secondary antibody, goat-anti-chicken (RRID:AB_10984880), diluted 1:500 in the blocking solution. After washing with PBS for 3 × 5 minutes, sections were incubated for 1 hour with an avidin-biotin complex (ABC Elite kit, Vectastain; Vector laboratories, CA). Sections were washed again with PBS for 3 × 5 minutes, before being incubated in DAB solution (0.5 mg/mL PBS). After 15 minutes, H_2_O_2_ was added to the DAB solution. The reaction was stopped after 3 minutes by washing in PBS (3 × 5 minutes). Sections were mounted onto slides gelatinized with 1% gelatin and 0.05% chromium potassium sulphate, dried, dehydrated by a series of alcohol concentrations and cleared using X3B. Lastly, slides were coverslipped using Safety Mount No. 4 mounting medium (Thermo Fisher Scientific, NSW, Australia).

### Digitization and organization of images

The immunostained brain sections were scanned with a Zeiss Axioscan Z1 scanner (Carl Zeiss MicroImaging, Jena, Germany), using a 20 × objective. The white balance was adjusted in the ZEN software (RRID:SCR_013672), and images were exported at full resolution (pixel width 0.22 µm) as lossless tiled TIFF images.

A series of pre-processing steps was implemented prior to sharing the images. First, images were renamed using a consistent naming scheme including the genotype, age group, sex, animal ID, and section number, separated by underscores (e.g. D1R_P70_M_C9_s001, D1R_P70_M_C9_s002, etcetera). Section numbers were selected to reflect serial order from rostral to caudal. If necessary, images were rotated and/or flipped horizontally to account for tilts and mirroring of sections during mounting. These pre-processing steps were performed using the Transform function in the Nutil software^18^ (v.403, RRID:SCR_017183). In rostral and caudal parts of the brain, where coronal sections include detached parts (such as the olfactory bulb, brain stem, and occipital cortical hemispheres), some section images include erroneously mounted parts that were not corrected (e.g. the brain stem being mirrored but not the cortical hemispheres). Such deviations are noted in the data descriptors provided with each dataset on EBRAINS.

Sections with poor tissue quality (defined as less than one half section being intact) or poor staining quality were excluded. Excluded sections retained their name in the serial order. The information on excluded sections are inferable from the names of the remaining sections, allowing their accountability in analyses. High-resolution images and atlas overlays were organized via the Navigator N3 data system^19^ at the University of Oslo and disseminated to an interactive web-microscopy viewer.

### Registration to reference atlas

To facilitate interpretation of the data and enable their use in brain-wide analyses, all images were spatially registered to three-dimensional reference atlases. For the late adolescent (P49) and adult (P70) age groups, we used the Allen Mouse brain Common Coordinate Framework (CCF, version 3, 2017 edition of delineations^13^, hereafter referred to as CCFv3-2017), which is based on a template from P56 mice. For the younger age groups, we used the developmental 3D brain atlases provided by Newmaster and colleagues^14^. These atlases were created by non-linear 3D warping of the delineations from the CCF to serial two-photon microscopy (STPT) templates from young brains, as described by Newmaster *et al*.^14^. Such STPT images from a population of brains were averaged to create templates for ages P7, P14, P21 and P28^14^. We used the P14 atlas for the material from the P17 group, and the P28 atlas for the material from the P25 and P35 groups.

To use these atlases for spatial registration of the 2D section images shared in the current study, we implemented each of the atlases by Newmaster and colleagues in the QuickNII tool (RRID:SCR_016854)^20^. The voxel resolution of these 3D volumes are 20 × 20 × 50 µm (non-isotropic). These were represented as isotropic voxels in QuickNII. While this does not affect the anatomical precision achieved with the spatial registration (the custom atlas maps are still valid), the coordinates from the volume do not represent realistic distances between landmarks in the brain. Prior to bundling each atlas with QuickNII, we adjusted some of the segmentations and their labels to ensure consistency with the adult CCFv3-2017. In the original label descriptions provided by Newmaster and colleagues^14^, atlas regions were not assigned colors. We therefore created a label set with color codes according to the CCF hierarchy.

All microscopic image series were spatially registered to the age-relevant atlases using QuickNII. This tool allows the user to cut the atlas in any plane of orientation, thus making it possible to account for deviations from the standard plane introduced during sectioning. For each series, such deviations were first identified by inspecting the relation of landmarks at dorsal and ventral positions (e.g. the nucleus accumbens and caudate putamen, hippocampus and optic tract) and across hemispheres. After defining the appropriate anteroposterior position for each section image, the customized atlas images were linearly scaled and rotated to match anatomical landmarks visible in each image. All registration results were validated independently by another investigator. The parameters specifying the spatial relationship defined between the images and the atlas were saved in an XML file and used for visualization of the custom-cut atlas plates in the high-resolution image viewer. More detailed notes on the spatial registration results for individual image series are provided in the EBRAINS data descriptors for each dataset.

### Curation and sharing of data through EBRAINS

The entire collection of images, hereafter referred to as the DOPAMAP collection, was shared via the EBRAINS Knowledge Graph as twenty datasets (Fig. 1, see “Data records” for details) together with metadata describing the subjects and methods used.

## Data Records

The DOPAMAP collection consists of twenty datasets^21–40^, each representing one combination of genotype (2 conditions), age (5 conditions) and sex (2 conditions) (Fig. 1; Table 1). These datasets are all hosted under unique DOIs and CC-BY licenses in the EBRAINS Knowledge Graph (Table 1). For each subject, 15 – 52 images are included; all datasets cover the rostral part of the forebrain, most extend caudally to the level of the anterior part of the hippocampus, and some to the level of the pontine nuclei. In addition to the option to download TIFF images at full resolution, a brain atlas viewer link is provided for each subject, through which the high-resolution images can be viewed with custom cut atlas plates overlaid.

**Table 1:** Overview of presented datasets.

Genotype	Age	Sex	Dataset DOI	# of subjects	# of images (average)	# of images (total)
D1R	P70	M	10.25493/AVRZ-4JB	9	37	334
		F	10.25493/5MXR-AW7	10	36	363
	P49	M	10.25493/GVFP-10X	9	34	303
		F	10.25493/31D4-SKG	6	43	255
	P35	M	10.25493/37DN-V7S	11	32	356
		F	10.25493/F3QR-M3S	8	35	283
	P25	M	10.25493/AMBF-6CV	8	39	311
		F	10.25493/C3A9-VVM	8	38	304
	P17	M	10.25493/TJK8-D7W	7	35	246
		F	10.25493/V09E-521	10	28	282
D2R	P70	M	10.25493/4DEB-5AJ	7	26	182
		F	10.25493/VTD0-D15	5	24	119
	P49	M	10.25493/ANPQ-05J	8	24	192
		F	10.25493/JJYX-T5R	8	23	187
	P35	M	10.25493/MFF5-KY5	7	26	184
		F	10.25493/1J85-QSK	8	22	176
	P25	M	10.25493/4G63-GDE	9	24	212
		F	10.25493/C3A9-VVM	6	22	130
	P17	M	10.25493/9DHD-DXZ	4	23	93
		F	10.25493/G5VR-63E	5	19	95
				**Total: 153**	**Avg: 30**	**Total: 4607**

Table showing the twenty datasets shared through the DOPAMAP project, each assigned a unique DOI. The table lists the number of subjects in each dataset, the average number of images available per subject and the total number of images for all subjects within each dataset.

## Technical Validation

### Visualization of D1- and D2 receptor positive cells

To visualize D1R and D2R positive cells, we used mice expressing EGFP under control of the D1R and D2R promoter, respectively. This allows visualization of cells expressing D1R and D2R mRNA, which likely is associated with protein expression. A potential pitfall of using such animals is that expression patterns may be altered by genetic manipulations, as seen in Kramer and colleagues’ study of homozygous Drd2-EGFP mice^41^. However, Kramer *et al*. used only homozygotic mice whereas our dataset only uses hemizygotic mice. While one laboratory reported a minor increase in basal locomotion in Drd2-EGFP hemizygotic mice compared to wild type mice^42^, another study assessing Drd1a- and Drd2-EGFP mice in three independent laboratories showed that these mice are indistinguishable from wild type mice in basal and cocaine-induced locomotion^43^. Based on such data, it is unlikely that the Drd1a- and Drd2-EGFP mice used in this study differs from wild-type mice in expression patterns, although this is yet untested. Because of the lack of validated and replicated antibodies for these proteins at the onset of this study in 2016 (however, see Stojanovic *et al*.^44^, where the specificity of one antibody for each protein was validated), the use of transgenic mice were deemed to be the best option for visualizing D1- and D2 receptor positive cells (see also Cullity *et al*.^10^ for a discussion on this decision). Particularly interesting future directions thus include the use of the antibodies that are recently and thoroughly validated^44^ to map D1- and D2 receptor positive cells to determine whether any differences exist between transgenic and wild-type mice, and to map expression in non-transgenic brains.

Mouse pups were genotyped by PCR, and only those hemizygous for the EGFP reporter gene were selected. The REDExtract-N-AMP^TM^ Tissue PCR Kit (Cat#: XNAT-100RXN, Sigma Aldrich, Castle Hill, Australia) was used to extract DNA from tail samples, and PCR was then performed using GoTaq® Green Master Mix (Cat#: M7122, Promega, Alexandria, Australia) according to the manufacturer’s protocols. Primers were purchased from Geneworks. The following forward and reverse primers were used:

Drd1a-EGFP forward primer: 5′-ACC GGA AGT GCT TTC CTT CTG GA-3′.

Drd1a-EGFP reverse primer: 5′-TAG CGG CTG AAG CAC TGC A-3′.

Drd2-EGFP forward primer: 5′-GAG GAA GCA TGC CTT GAA AA-3′.

Drd2-EGFP reverse primer: 5′-TGG TGC AGA TGA ACT TCA GG-3′.

### Section quality and coverage

**Fig. 2:**
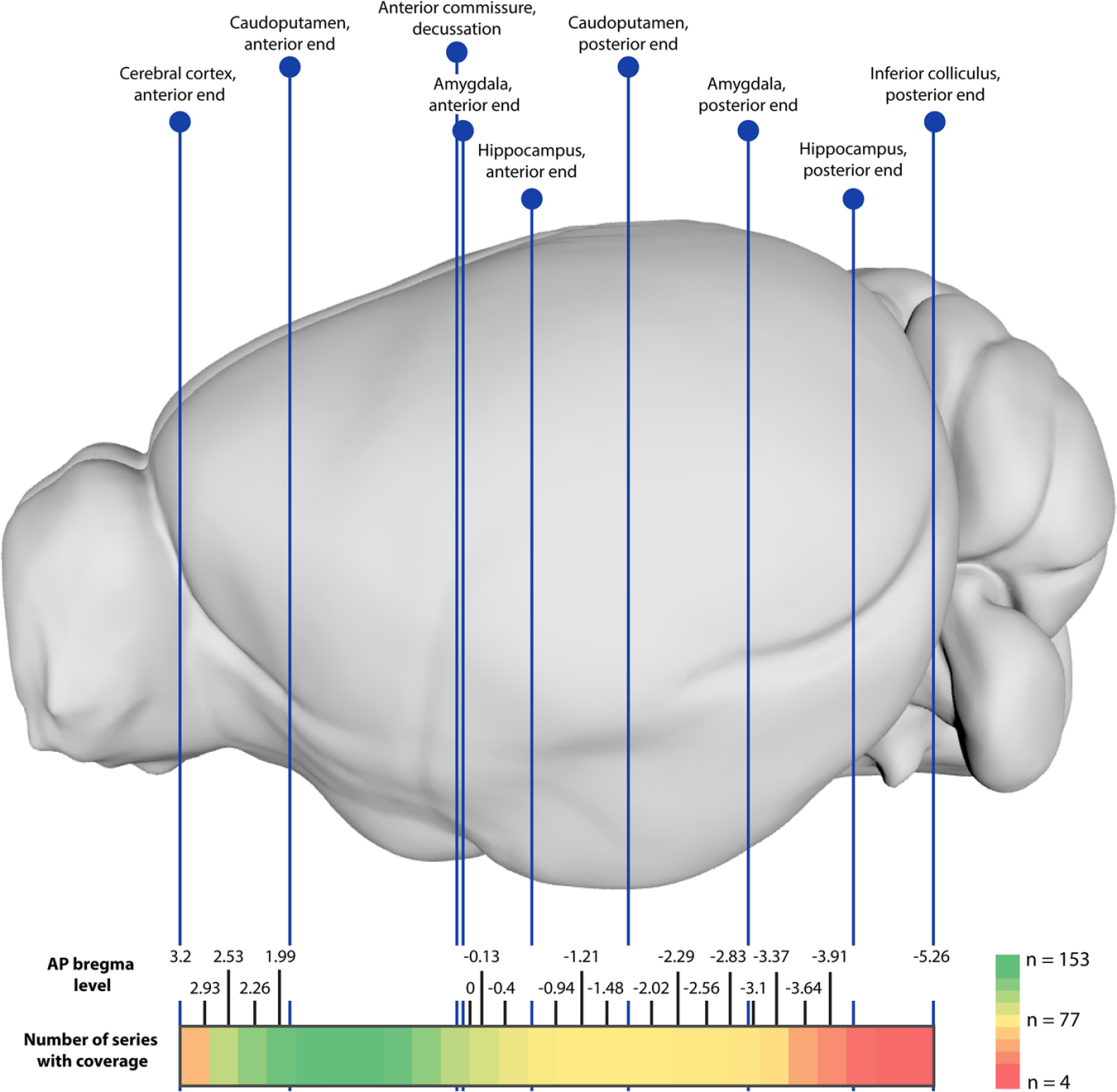
Coverage of the image series in the DOPAMAP project. The upper panel shows a lateral view of the CCFv3-2017, with the coronal level of key anatomical landmarks indicated. The number of series within the DOPAMAP project covering various coronal levels across the brain are indicated in the colored bar below, from red (very few series) to green (all series). Anteroposterior bregma level coordinates are extracted from the adult stereotaxic atlas by Franklin and Paxinos (version 3) for reference, coordinates for the corresponding landmarks in younger brains might differ from these.

Free-floating immunohistochemistry, as used here, involves several steps that may lead to damage or distortion to tissue sections. Common problems include holes or tears in a section, tissue folding, as well as displacements of different brain parts relative to each other due to errors during the mounting process. We here evaluated the quality of the sections for each series. To assess section quality, we designed a semi-quantitative scoring system ranging from 1 (“Almost all sections have maximum acceptable damage and poor mounting”) to 5 (“Almost all sections are whole and intact, with minimal damage, and good mounting”). We defined minimal damage as less than approximately 10% of the section being damaged, and poor mounting as mounting errors that cannot be resolved by rotating the image as a whole (e.g. individual parts of the tissue section being flipped horizontally or vertically, or hemispheres/brain parts being incorrectly combined). While such damage should ideally be avoided, it usually affects only small parts of a section with the remainder of the image still containing highly valuable information. We also evaluated how much of the brain, from anterior to posterior, each series covered (referred to as *coverage*). To evaluate this, we noted the approximate anteroposterior bregma location of the first and last section, and the number of sections included in each series. The average quality score was 3.6 (range = 1–5, SD = 1.05). The average number of sections was 30 (range = 15–52, SD = 11.4). All series covered the part of the rostral forebrain from the anterior end of the caudate putamen, to approximately the level of the anterior commissure decussation (Fig. 2). Most series also covered the anterior end of the hippocampus, and approximately half the series extended throughout the thalamus. A small proportion of the series also covered the anterior end of the cerebral cortex and the caudal end of the inferior colliculus. A detailed overview of the coverage for each series is given in Supplementary File 1.

## Usage Notes

### General value of the DOPAMAP collection

The DOPAMAP collection provides a uniquely rich source of data on D1R and D2R positive cells in developing and adult mouse brains. Until now, our collective knowledge about these cells has been fragmented across publications focusing on one or a few brain regions^10–12,45,46^. While many detailed studies have been performed in the striatum^11,47^, other regions are understudied, especially in developing female mice.

Spanning five age groups and including both male and female subjects, the DOPAMAP collection will be an important resource for any researcher interested in studying any forebrain region with D1R and D2R positive cells. Researchers can easily look up microscopic images and confirm presence and study distribution of these cells in their regions of interest. New studies can be designed based on the neuroanatomical presence of dopamine receptors in regions such as the claustrum that are poorly understood in their dopaminergic function across development. Users interested in age and sex differences can download and utilize the data in novel analyses. Access to raw image collections is especially relevant for computational researchers, who are typically interested in analyzing large datasets without having to perform new experiments. The stereological data provided for selected brain regions in the related publication^10^ adds valuable quantitative information about the cells visualized in this material, which can be complemented by future quantifications in additional brain areas, e.g. by semi-automated analyses^48,49^. The high resolution of the images also permits investigations of morphological parameters, such as size and shape of neurons, which could be useful to identify different cell types across brain regions. Thus, we believe that the DOPAMAP collection can be a valuable resource for several types of neuroscientists. Technical details needed to support re-use of the material in new analyses are provided in the EBRAINS data descriptors for each dataset. Below, we give some example use cases to further elaborate on how the collection may be used in the future.

### Use cases

#### Finding and exploring the data

**Fig. 3:**
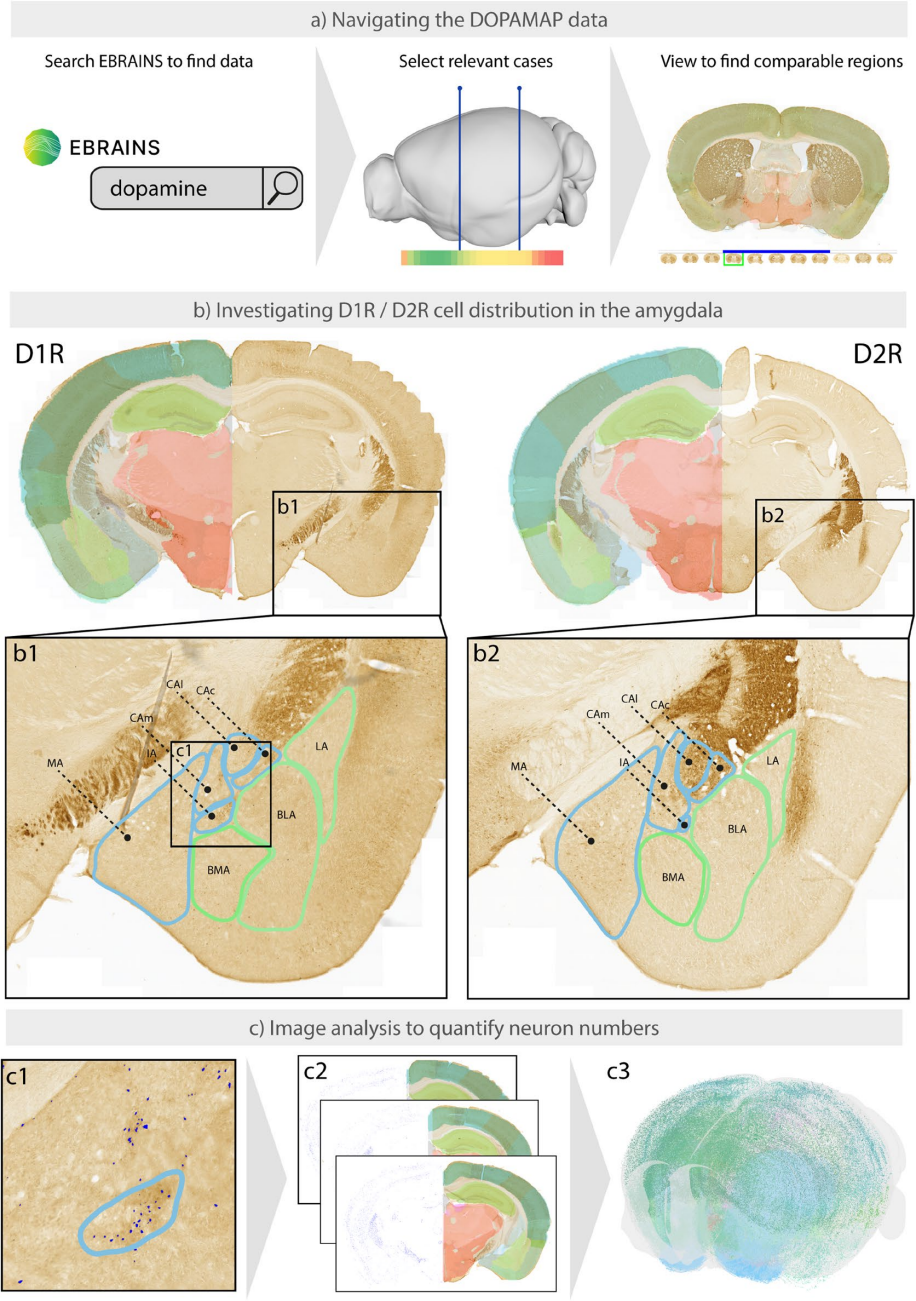
Use cases for the DOPAMAP data. Researchers may use the DOPAMAP data to explore the expression of D1R and D2R cells in their regions of interest (**a**), or to perform region-wise or brain-wide analyses to compare subjects by sex, age or genotype (**b,c**). (**a**) Finding and exploring the DOPAMAP data through the EBRAINS Knowledge Graph. (**b**) Example comparison of two DOPAMAP datasets. Images shown here are image s034 from series C22 (D1R male subject) and s033 from series D146 (D2R male subject), at approximate Bregma −1.88. (**c**) Example analysis of the dopamine 1 receptor positive cells in subject C22 using the QUINT semi-automatic image analysis workflow^48^. Cells are segmented using the ilastik software (c1), and the resulting segmentation images are combined with the atlas maps (c2) to generate a 3D map of the cells (c3). Abbreviations: BLA, basolateral amygdala; BMA, basomedial amygdala; CEAc, central amygdala, capsular part; CEAm, central amygdala, medial part; CEAl, central amygdala, lateral part; IA, intercalated amygdala; LA, lateral amygdala; MEA, medial amygdala.

A researcher exploring the role of D1R and D2R cells in the adult amygdala wants to investigate the relative distributions of these cells across detailed sub-regions of the amygdala. To search for relevant public datasets, the researcher searches for “dopamine” and “mouse” in the free-text field (Fig. 3a) at EBRAINS Knowledge Graph (https://search.kg.ebrains.eu/?facet_type[0]=Dataset). The search returns the DOPAMAP datasets as the most relevant results. Upon clicking any of these datasets, a landing page for the dataset is shown. The landing page includes an overview of the dataset, a brief data descriptor on the data acquisition and file structure, citation information, links to download or view the data online, and information about the subjects and tissue samples. From the landing page of any DOPAMAP dataset, the researcher can also access the DOPAMAP project landing page, where all the datasets are listed.

Supplementary document 1 contains an expansion of Fig. 2, giving a complete overview of the brain coverage of each series in the DOPAMAP collection, with indications of the brain regions and bregma levels covered by each case. The researcher could use this document to identify cases covering a specific brain region. Example series covering the entire amygdala includes case C22 for D1R and D146 for D2R (both male subjects), images from which are shown in Fig. 3b. The viewer links provided on the dataset landing pages (available from the “View data” option in the side bar) can then be used to closely examine the series of interest. Navigation to the region of interest and inspection of the patterns of distribution is enabled by using the atlas maps as a guide, with region names according to the atlas appearing when hovering over the atlas overlay with the mouse cursor. The atlas overlay can be toggled on and off by using the “Atlas opacity” slider; sliding the bar to the far right results in atlas regions being shown as outlines only, while sliding it to the far left removed the atlas overlay completely. The microscopic image can be zoomed and panned to inspect the staining patterns in regions of interest. In the case of the amygdala, heavy staining indicative of abundant D1R positive cells is observed in the intercalated nucleus. By contrast, the central, basomedial and basolateral amygdala display very few cells (except for a few specific localized spots), while the lateral amygdala is devoid of staining (Fig. 3b1). While the immediate observations are similar for D2R cells, the intercalated nucleus displays no appreciable staining of D2R compared to D1R, while the central nucleus has a quite dense population of D2R cells, most notably in the capsular part (Fig. 3b2).

### Quantitative analysis to compare subjects by sex, age or genotype

A key opportunity arising from the publication of the DOPAMAP data is the possibility for quantitative analysis of the cell types across sex, age or genotype. Although performing such analyses across the DOPAMAP collection is beyond the scope of the present study, Fig. 3c exemplifies for one case how cells visible in the images may be segmented, quantified and visualized in 3d. Researchers wanting to determine whether numbers and distributions of the two cell types differ, or whether there are differences between males and females or across age groups, may download the original TIFF images to do so. There are several software solutions for segmenting features of interest from microscopic images (Fig. 3c1-2), e.g. ImageJ^50^, ilastik^51^, QuPath^52^, and a range of other tools. To combine segmented images with anatomical information from the reference atlases (Fig. 3c1-2) and visualize the extracted coordinates in 3D (Fig. 3c3), users may employ the QUINT workflow^48^. This workflow combines three open-source tools with graphical user interfaces, enabling efficient quantitative analysis of the data across entire image series. Figure 3c shows the applications of the QUINT workflow to an example case (ID C22) from the adult male D1R group. The detailed procedure for applying the QUINT workflow to immunohistochemical material has been described in previous publications^48,49^.

### Comparing expression patterns with those of other markers

**Fig. 4:**
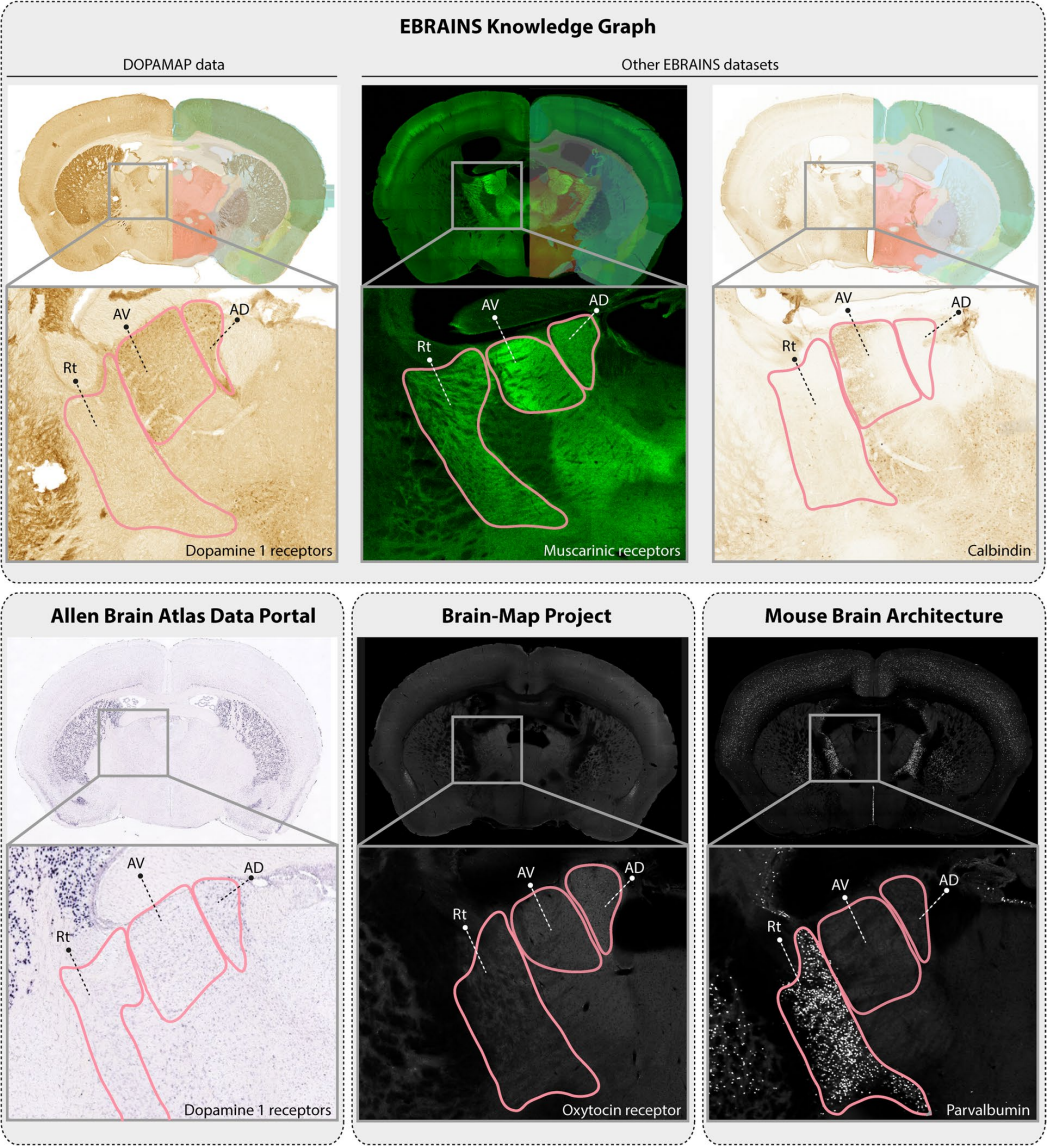
Comparing DOPAMAP data to images from different repositories. Several public repositories, including EBRAINS, contain similar data as the ones presented here for other markers. The images exemplify available data on the distribution of muscarinergic receptors and calbindin (from other datasets in the EBRAINS Knowledge Graph), D1R mRNA (from the Allen Brain Atlas Data Portal), oxytocin receptors (from the Brain-Map Project) and parvalbumin (from the Mouse Brain Architecture Project). The user can inspect and compare images from different repositories side-by-side, aided in anatomical interpretation by available atlas maps and/or other resources such as the EBRAINS Interactive Atlas Viewer and QuickNII^20^. The magnified images shows an example of such a comparison for three sub-regions of the thalamus. Examples shown: dopamine 1 receptor data^21^ (EBRAINS), subject C22, image s027; muscarinic receptor data^54^ (EBRAINS), subject G06, image s154; calbindin data^53^ (EBRAINS), subject mouse11, image s169; dopamine 1 receptor data (Allen Brain Atlas Data Portal), experiment no. 352, image 20; oxytocin receptor data (Brain-Map Project), OTR-eGFP BAC transgenic reporter mice male P56, individual image number not available; parvalbumin data (Mouse Brain Architecture), sample ID OST_U01_140602, image 115. Abbreviations: AD, anterodorsal nucleus; AV, anteroventral nucleus; Rt, reticular nucleus.

Researchers interested in comparing the expression patterns of D1R or D2R positive cells with those of other cell types or markers, could compare the DOPAMAP data with other public data. In the EBRAINS Knowledge Graph, other similar datasets can be identified by using the faceted search. Restricting the species filter to “Mus musculus” and the methods filter to “Immunohistochemistry” and “Brightfield microscopy” yields 49 datasets, e.g. showing the distribution of calbindin neurons^53^, parvalbumin neurons^49^, and muscarinic receptors^54^ (Fig. 4, top panel). Additional resources with similar data types include the gene expression database from the Allen Institute (http://mouse.brain-map.org/)^55,56^, the cell type repository of the Mouse Brain Architecture project (www.brainarchitecture.org/), and the Brain-Map Project showing oxytocin receptor expressing cells in the developing mouse brain (https://kimlab.io/brain-map/ot_wiring/)^14^. Figure 4 (bottom panel) shows example images from series available from each of these resources. Similar to EBRAINS, all of these projects provide viewers where the user can easily navigate and inspect data. Custom cut atlas overlays from the Allen Mouse brain Common Coordinate Framework allows rapid identification of regions of interest, and facilitate comparison across EBRAINS datasets. Image series from the Allen Institute are displayed side-by-side with corresponding atlas maps, while the Mouse Brain Architecture project provides the anteroposterior bregma level for each image. These features can also be used as a starting point to identify similar positions across resources. Where atlas maps are not provided, online atlas viewers such as the EBRAINS Interactive Atlas Viewer (https://interactive-viewer.apps.hbp.eu/#/) may be used to navigate to similar positions in the Allen Mouse brain Common Coordinate Framework. However, if the sections to be interpreted are cut with a deviation from the coronal plane (as for the example series from the Allen Institute shown in Fig. 4), a single atlas plane is insufficient for accurate anatomical analysis. In these cases, atlas registration tools such as QuickNII^20^ can be used to create custom cut atlas maps for a given section image.

### Identifying target areas for intracranial infusion with D1R or D2R agonists/antagonists

Researchers interested in the functional role of D1R and D2R neurotransmission in different brain loci could use the DOPAMAP collection to design studies. Intracranial injections of selective D1- or D2-receptor agonists are often used in such studies to investigate the significance of signaling through these receptors, and the data presented here can be used to identify suitable stereotaxic target areas. In particular, observed differences in cell density along the anteroposterior axis of different brain regions may provide useful guidance to where such injections should be made. Brain regions that are particularly devoid of D1R and D2R cells are also useful to identify as negative control regions. Sex- and age-specific information from DOPAMAP is particularly helpful in this regard for those interested in the behavioral consequences of dopamine signaling in certain brain regions in different populations. Even if dopamine signaling is not of interest, DOPAMAP images provide clear landmarks for most brain regions that can be targeted for intracranial surgery. Because brain atlases with stereotaxic coordinates do not exist for juvenile and adolescent rodents, researchers are often forced to guess and conduct multiple pilot surgeries to determine appropriate coordinates^57–59^. DOPAMAP can better estimate coordinates by comparing side to side an adult target image and juvenile/adolescent target image to determine the medial-lateral and dorsal-ventral adjustment typically required in juvenile intracranial surgery. In addition, the target region image distance from 0 bregma can also be counted to adjust the anterior-posterior coordinates.

## Supplementary information

Supplementary File 1

## Data Availability

We used the Zen software (RRID:SCR_013672) to export TIFF images from the original slide scans. Nutil v.403^18^ (RRID:SCR_017183) was used for preprocessing of images. QuickNII v2.2^20^ was used for spatial registration of images (RRID:SCR_016854).
