# Supplementary figures and images for: DOPAMAP, high-resolution images of dopamine 1 and 2 receptor expression in developing and adult mouse brains
Source: Sci Data. 2022 Apr 19;9:175. doi: 10.1038/s41597-022-01268-8 (PMC9018709; doi:10.1038/s41597-022-01268-8)

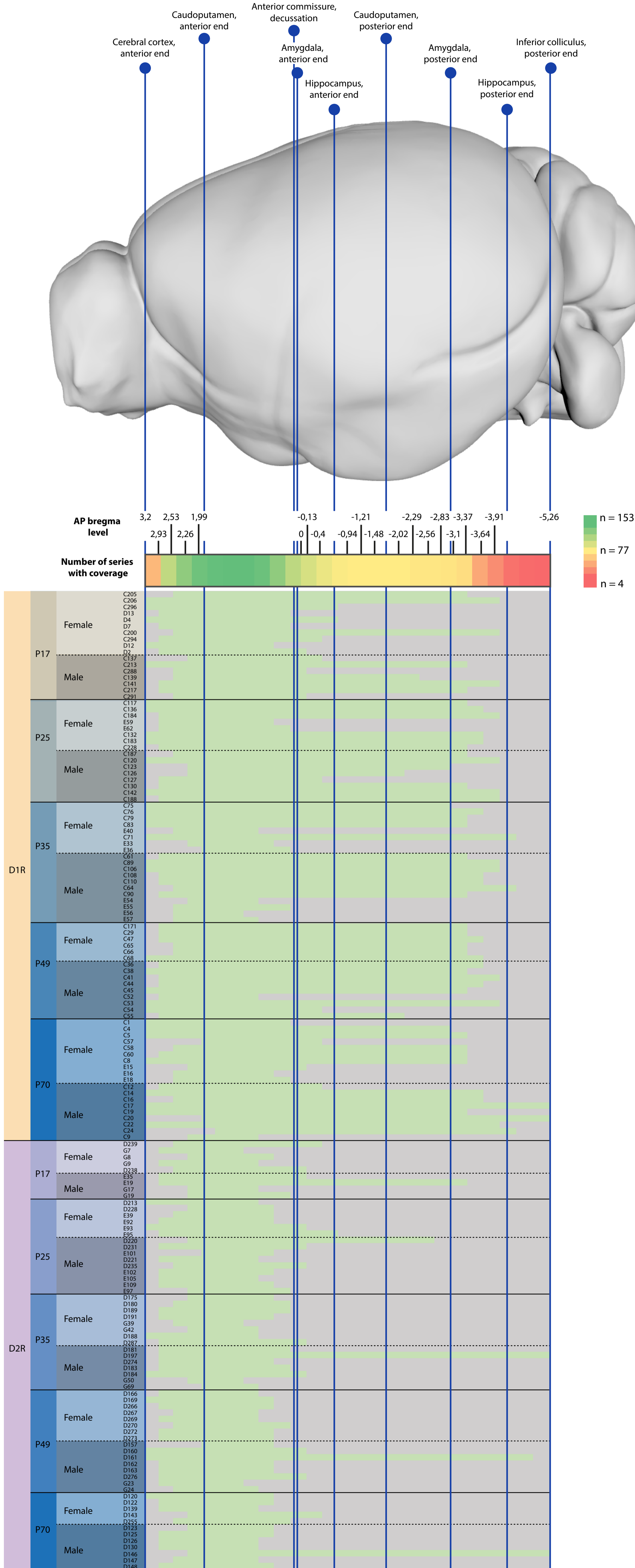

Supplement: Supplementary file 1 [file 41597_2022_1268_MOESM1_ESM.pdf]
